# Bilateral breast metastases from rectal malignant melanoma: case report and literature review

**DOI:** 10.3389/fonc.2025.1565838

**Published:** 2025-08-18

**Authors:** Hui Liu, Qian Liu, Linshen Zhang, Qinghua Liu, Hao Cheng, Jian Liao, Zhaohong Chen, Dingyu Lu

**Affiliations:** ^1^ Department of Oncology, Deyang People’s Hospital, Deyang, Sichuan, China; ^2^ Department of Pathology, Deyang People’s Hospital, Deyang, Sichuan, China; ^3^ Molecular Oncology Laboratory, Technical University of Munich and Klinikum Rechts der Isar, Munich, Germany; ^4^ Intensive Care Unit, Deyang People’s Hospital, Deyang, Sichuan, China

**Keywords:** rectal malignant melanoma, secondary malignant tumor of the breast, malignant melanoma, breast metastasis, case report

## Abstract

Rectal melanoma is an extremely rare and highly aggressive disease, and rectal melanoma metastasis to the breast is rare. This is a 48-year-old female who presented with rectal melanoma carcinoma Following the diagnosis of locally advanced rectal cancer (TxN3M0), she received chemotherapy, immunotherapy with radiotherapy, and metastases to the lung, bone and vulvar developed during the therapy. After one year of treatment, the patient developed bilateral breast metastases. Then breast site was identified using the gene expression assay which showed KIT mutation, indicating sensitivity to imatinib, so the patient had used imatinib for 2 months. But then the patient started to take Chinese medicine for personally reason. Sadly, the patient passed away 22 months after initial diagnosis due to rapidly progressive disease. This case showed a rare presentation of rectal melanoma metastasis to the breast. To our knowledge, this is also the first report of rectal melanoma metastasis to bilateral breasts.

## Introduction

Rectal melanoma is an extremely rare and highly aggressive disease, and the prognosis is reported to be extremely poor. The most common symptom in patients with rectal malignant melanoma is difficulty in defecation (63%), followed by rectal bleeding (50%). The most common sites of distant metastasis are the liver and the lungs owing to anatomical features of the venous drainage system of the rectum. In most studies, metastatic spread to the breast from extramammary sites has an incidence of 0.5–3% (1). Of mammary malignancies, the primary lesions are derived mainly from the contralateral breast, and metastatic rectal cancer is rare (2).

In this paper, we report the case of a 48-year-old woman with bilateral breast metastasis derived from rectal melanoma. The patient presented with rectal bleeding, with the diagnosis of locally advanced rectal cancer (TxN3M0), Treatment strategies included chemotherapy, immunotherapy, radiotherapy, and targeted therapy. Finally, the patient achieved a total survival of 22 months.

To our knowledge, this is the first case of bilateral breast metastasis arising from rectal melanoma. This case report aims to enhance the clinicians’ recognition and awareness of the invasiveness and occult metastasis of rectal melanoma.

## Case report

**Figure 1:**
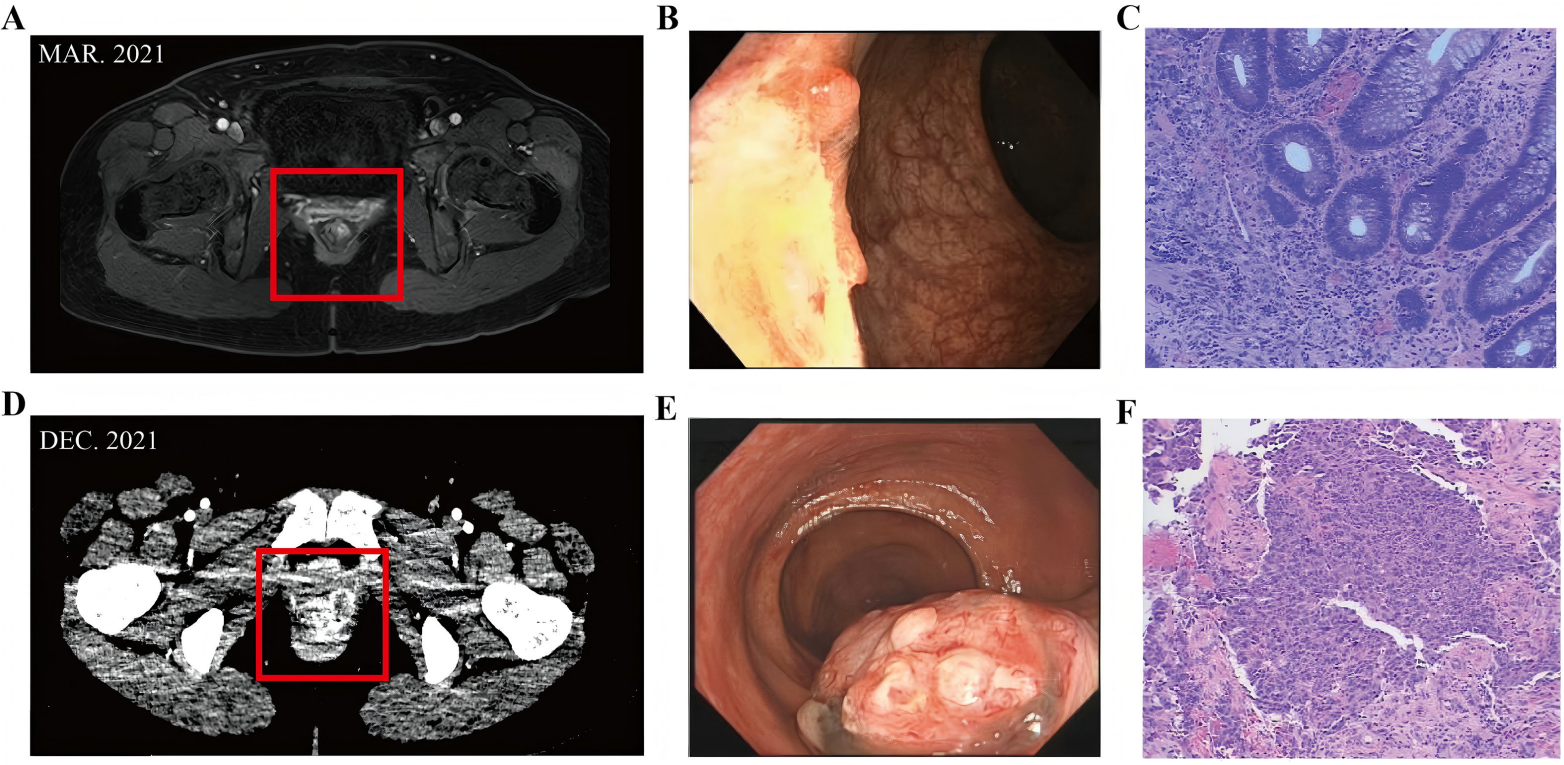
**(A)** Enhanced pelvic Magnetic Resonance Imaging (MRI) in outpatient showed an abnormal signal focus on the anterior wall of the lower rectum, locally involving the anal canal. **(B)** Colonoscopy indicated a lesion near the rectum within the anal canal, considered ulcerative or other pathology. **(C)** Inflammatory granulation tissue interspersed with atypical epithelioid cells. **(D)** Enhanced pelvic CT suggested lower rectal cancer. **(E)** The results of the colonoscopy indicated a rectal-anal neoplasm with bleeding. **(F)** Histopathological examination confirmed melanoma.

In March 2021, a 48-year-old woman presented to us with intermittent rectal bleeding for three months. Her first enhanced pelvic Magnetic Resonance Imaging (MRI) in outpatient (**Figure 1**) showed an abnormal signal focus on the anterior wall of the lower rectum, locally involving the anal canal, suggesting possible rectal carcinoma; multiple small lymph nodes were visible in the mesorectum, bilateral iliac vascular regions, and inguinal areas. Pelvic-enhanced computed tomography (CT) findings were consistent with MRI. The initial colonoscopy indicated a lesion near the rectum within the anal canal, considered ulcerative or other pathology (**Figure 1**). Histopathologic examination of two of the four core biopsy samples from the rectum showed chronic inflammation with crypt abscesses and pigment deposition, and the other two contained inflammatory granulation tissue interspersed with atypical epithelioid cells (**Figure 1**). Further tissue sampling was recommended. Routine blood investigations were unremarkable, including serum carcinoembryonic antigen (CEA) and carbohydrate antigen (CA) level, but fecal occult blood was positive. However, the patient refused to undergo further colonoscopy and therapy.

Nine months later (December 2021), the patient returned to us with recurrent rectal bleeding. The results of the colonoscopy indicated a rectal-anal neoplasm with bleeding (**Figure 1**), and histopathological examination confirmed melanoma (**Figure 1**). Rectal immunohistochemical detection: CK EMA (-) (-), Vimentin (+) S - 100 (+) oven HMB 45 (+) - (+) the SOX Melan - A - 10 (+) about (-) CK56 P40 (-) (-) Ki - 67 (+ 70%), in accordance with melanoma. Enhanced pelvic CT suggested lower rectal cancer (**Figure 1**), the maximum layer of the rectal lesion is approximately 5.78cm to 3.75cm, and found four abnormal pelvic lymph nodes; Enhanced chest and abdominal CT scans showed no abnormalities. On physical examination, a 2-cm hard, poorly mobile mass was palpated at 3 cm from the anal verge, with smooth rectal mucosa and minor Bleeding upon finger withdrawal.

The patient was diagnosed with rectal melanoma with pelvic lymph node metastasis (TxN3M0, stage IIIC) by Multidisciplinary team (MDT) according to the 2021 Chinese Society of Clinical Oncology (CSCO) guidelines.

**Figure 2:**
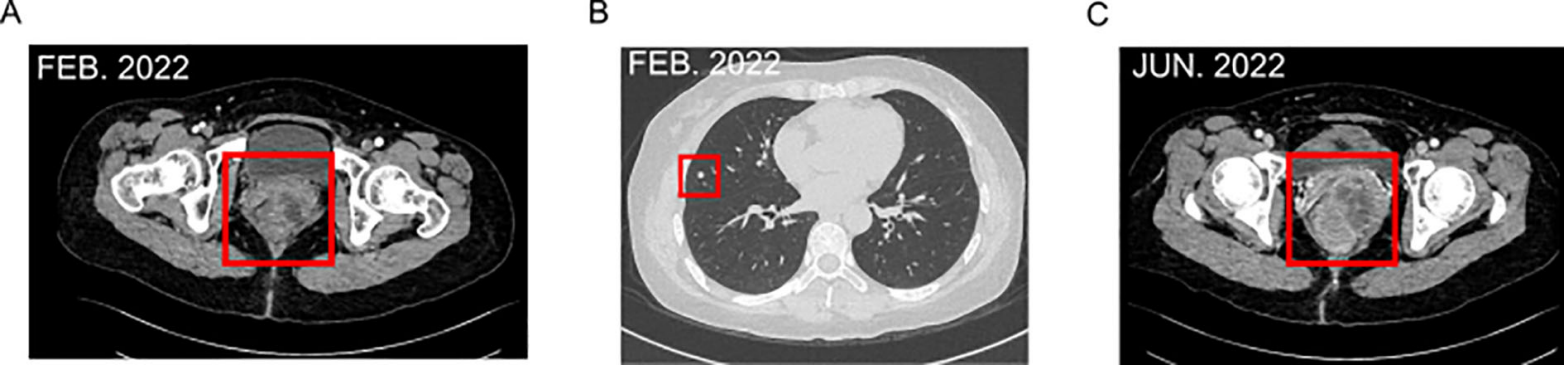
**(A)** Enhanced CT scan on February 17, 2022, the lower rectal lesion and pelvic lymph nodes had significantly enlarged, with invasion into the vagina. **(B)** Solid nodules in the right upper and middle lobes of the lung. **(C)** June 2022, Enhanced CT showed a reduction in rectal lesions and pelvic lymph nodes.

Then the patient completed two cycles of the same systemic chemotherapy combined with immunotherapy (dacarbazine and sintilimab injection). Then a follow-up enhanced CT scan on February 17, 2022, the maximum layer of the rectal lesion is approximately 7.56cm to 5.4cm, showed tumor progression: the lower rectal lesion and pelvic lymph nodes had significantly enlarged (**Figure 2**), with invasion into the vagina. Solid nodules in the right upper and middle lobes of the lung suggested metastases (**Figure 2**). Efficacy was evaluated as progressive disease (PD).

Subsequently, the patient received two cycles of chemotherapy with albumin-bound paclitaxel and carboplatin injection (February 24 and April 2, 2022). In April 2022, intensity-modulated radiotherapy was applied to the rectal melanoma with a total dose of 48 Gy in 24 fractions. By June 2022, the patient felt back pain [Numerical Rating Scale(NRS) score of 2]. Enhanced CT showed a reduction in rectal lesions and pelvic lymph nodes (**Figure 2**), the maximum layer of the rectal lesion is approximately 6.1cm to 3.96cm, with stable lung metastases. However, new bony destruction was observed in the right fourth rib, T4 vertebral pedicle, and transverse process, suggesting metastasis. Bone scans confirmed possible tumor bone metastases in the right fourth and fifth ribs and T4 vertebra. Efficacy was again evaluated as PD.

Further treatment included zoledronic acid for bone destruction, and genetic testing. Throughout radiochemotherapy, the patient experienced grade III hematological toxicity but no allergic reactions, liver or kidney damage, or severe gastrointestinal side-effects.

**Figure 3:**
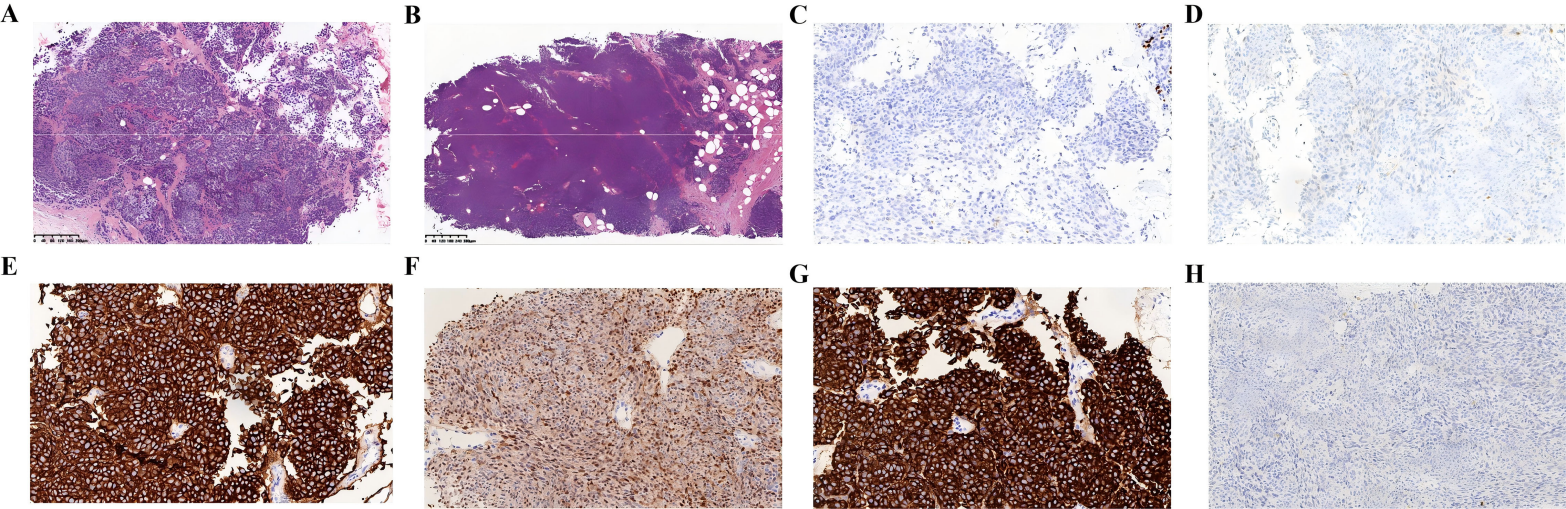
Pathology indicated malignant melanoma based on immunohistochemistry. **(A)** Right breast 9C. **(B)** Lleft breast 11C. **(C)** ER(-). **(D)** PR(-). **(E)** HMB15(+). **(F)** S-100 (+). **(G)** Melan-a (+). (H) CK (-). [**(A–G)** ×200 magnification].

In July 2022, the patient presented to us with a palpable mass in her bilateral breast, a mass of about 1.2 to 1.5 cm was palpated in the outer upper quadrant of the right breast, and a mass of about 0.8 to 1.0 cm was palpated in the inner upper quadrant of the left breast. Both were tender and had poor mobility, an ultrasound-guided biopsy of bilateral breast nodules (categorized as BI-RADS 4C) revealed malignancy. Pathology (right breast 9C, left breast 11C) indicated malignant melanoma based on immunohistochemistry: tumor cells were P63 (-), P40 (-), Vimentin (+), CK5/6 (-), CK (-), EMA (-), SMA (-), Syn (-), CgA (-), CD56 (+), Calponin (-), PR (-), ER (-), P120 (membrane +), E-Cadherin (+), CerbB-2 (0), CD10 (focal +), S-100 (+), Melan-A (+), HMB45 (+), and Ki-67 (+, approximately 70%) (**Figures 3A–H**). At the same time, Genetic testing revealed a KIT mutation, indicating sensitivity to imatinib. So extra oral imatinib monotherapy began in August 2022 and continued for two months. Then the patient stopped taking imatinib for personal reasons and chose to take traditional Chinese medicine on her own. Sadly, it was confirmed that the patient had passed away in October 2023. The total survival period was 22 months, with no progression-free survival achieved.

## Discussion

Rectal melanoma is an exceedingly rare condition, distinct from melanoma, and is associated with significantly poor outcomes. Metastatic spread to the breast from extramammary sites is very rare. Rectal melanoma metastasizing to the breast is an extremely rare event. We searched the PubMed database for case reports between 1999 and 2024 using the keywords ‘breast metastasis’ and ‘rectal cancer’. Including our report, 16 reports of rectal metastasis to the breast have been documented to date (**Table 1**). According to the previous reports, the majority of the patients were women with the primary tumor in the rectum pathologically confirmed poorly differentiated adenocarcinoma. The patient in our case was rectal melanoma, which is different from the others.

**Table 1:** 16 reports of rectal metastasis to the breast.

			Primary rectal cancer				Breast metastasis		
Number	Author/Year	Sex/Age	Location	pathology	organ metastasis	Management	Time of detection of breast metastases	Laterality	Size (cm)
1	Lal, R. L (3)./1999	F/69	Rectum	Moderately differentiated mucinous adenocarcinoma	Breast, Skin, liver, lung, brain	Surgery + chemotherapy	1 year	Left	NS
2	David, O (4)./2002	F/42	Rectum	Small cell undifferentiated carcinoma	Breast, Posterior cervical lymph node	NS	2 years	Bilateral	NS
3	Mihai, R (5)./2004	F/53	Rectum	Poorly differentiated adenocarcinoma	Breast, Lung, skin	Chemotherapy	5 years	Left	1
4	Li (6)/2009	F/54	Rectum	invasive poorly differentiated adenocarcinoma	Breast	Surgery+ Chemotherapy + radiotherapy	NS	Right	3
5	Makhdoomi R (7)/2013	F/28	Rectum	adenocarcinoma with signet-ring differentiation	Breast	Surgery	9 months	bilateral	3*2/2 *1
6	S.S. Ahmad (8)/2019	F/43	Rectum	poorly differentiated adenocarcinoma	Breast	Chemotherapy +targeted therapy	NS	Right	2.6
7	Cheng (9)/2020	M/57	Rectum	poorly differentiated adenocarcinoma	Breast,	chemotherapy	8 months	Right	3 * 3.9 * 1.3
8	Hiroko Hasegawa (10)/2020	F/67	Rectum	adenocarcinoma	Breast	Surgery + chemotherapy	1 year	Left	1* 1
9	Ye/2020	F/49	Rectum	Poorly differentiated adenocarcinoma	Breast, Lung, liver, bone	Radiotherapy + targeted therapy	3years	Left	2.5*2
10	Wang (11)/2021	M/38	Rectum	NS	Breast	Surgery + chemotherapy	7 years	right	6.0*6.2 *3.3
11	Wang (12)/2021	F/59	Rectum	mucous adenocarcinoma and signet ring cell carcinoma	Breast	neoadjuvant radiotherapy and chemotherapy.	16 months	left	1.9* 1.5
12	Dai (13)/2022	F/45	Rectum	Poorly differentiated signet ring cell carcinoma	Breast, lung	Surgery + chemotherapy + targeted therapy	3 years	Left	1.6 *1
13	Han (14)/2023	F/26	Rectum	poorly differentiated adenocarcinoma	Breast, vulvar, lung	Surgery + neoadjuvant chemotherapy	9 months	Right	NS
14	Xu (15)/2023	F/41	Rectum	Rectal low-differentiated adenocarcinoma	Breast	Surgery + chemotherapy	18 months	Left	15*10
15	Gouli (16)/2024	F/67	ascending colon	Poorly differentiated adenocarcinoma	Breast,	Surgery +Chemotherapy	NS	Left	1.4*0.8
16	Current case	F/48	Rectum	melanoma	Breast, Posterior cervical, vulvar, lung, bone	Chemotherapy + immunotherapy	1 year	Bilateral	

NS, No Size.

Treatment strategies remain controversial due to the disease’s rarity. Rectal melanoma is less responsive to chemotherapy and radiotherapy. Surgery is considered the most effective treatment, with options like abdominoperineal resection (APR) and wide local excision (WLE) (17). However, in our case, the patient did not undergo surgery. The patient agreed to undergo treatment only 12 months after symptoms of rectal bleeding. By the time the disease was diagnosed, the patient was already in the locally advanced stage, with lymph node metastasis. The optimal window for surgery had been missed. Metastases from the rectum to the breast were first reported by Lal in 1999 (3). In accordance with the previous studies in **Table 1**, half of the cases metastasized to the left breast. Some studies suggested this laterality may indicate the possibility of lymphatic preponderance instead of hematologic routes, but could not find any relation between this preference and other clinicopathologic features (13, 18). In our case, the patient developed bilateral breast metastases. The lymphatic pathway cannot be ruled out as an underlying mechanism of breast metastasis from rectal melanoma (13). More studies and observations about this phenomenon are necessary. But, we can make sure that metastasis to the breast from rectal cancer signifies disseminated metastatic disease or a highly aggressive tumor.

Overall melanoma-specific survival was poor irrespective of gender or ethnicity (19), and the reported survival time is 15 months (20). In our case, the patient’s total survival is 22 months. The patient missed the optimal surgical window and failed to receive treatment on time, contributing to a poor prognosis. Despite employing multiple therapies, the patient exhibited rapid disease progression and poor survival outcomes. Recent studies show an increase in immunotherapy usage for rectal melanoma (21). However, this patient showed poor response to all treatments. The result of Immunohistochemistry revealed high tumor cell proliferation (Ki-67 positivity ~70%), indicating aggressive tumor growth. As in the cases in **Table 1**, our patient succumbed to the aggressive course of the disease after the diagnosis of the primary tumor. So, early diagnosis and treatment are crucial for better outcomes in such rare cancers.

## Conclusion

Rectal melanoma is an extremely rare and aggressive cancer with non-specific clinical and gross manifestations. Early diagnosis and standardized treatment are crucial for improving prognosis. Clinicians should consider the possibility of breast metastasis, when a breast tumor is incidentally discovered during the treatment process.

## Data Availability

The original contributions presented in the study are included in the article/supplementary material. Further inquiries can be directed to the corresponding author.
